# Heating the land cools the eastern and equatorial Pacific

**DOI:** 10.1126/sciadv.aeb7004

**Published:** 2026-06-03

**Authors:** Moritz Günther, Sarah M. Kang, Yohai Kaspi

**Affiliations:** ^1^ Climate Dynamics Department, Max Planck Institute for Meteorology, 20146 Hamburg, Germany.; ^2^ Department of Earth and Planetary Science, Weizmann Institute of Science, Rehovot 7610001, Israel.

## Abstract

The unresolved recent multidecadal cooling of the eastern Pacific and its uncertain future remain key puzzles in climate dynamics. We propose the land-sea heating contrast as a potential driver of this phenomenon. To test this, we amplify the land-sea contrast by quadrupling carbon dioxide (CO_2_) only over land in a global coupled climate model. We show that this causes a pronounced transient cooling of the eastern and equatorial Pacific. A transient 1%-per-year CO_2_ increase over land produces decadal cooling, suggesting relevance to the observed eastern Pacific cooling. Targeted simulations with locally increased CO_2_ concentrations over different land regions reveal three drivers of this cooling: a northward intertropical convergence zone shift, a westward convection shift over the western Pacific, and strengthened subtropical highs from Rossby waves. These insights recast the commonly used ocean dynamical thermostat mechanism as a feedback rather than a driver of transient cooling and contribute to understanding and predicting Pacific temperature patterns.

## Teaser

Land-sea temperature contrasts in response to CO_2_ forcing contribute to the cooling of the eastern and equatorial Pacific.

## INTRODUCTION

Despite global warming, the southeastern Pacific Ocean has cooled in recent decades (*1*, *2*), and the equatorial Pacific zonal sea surface temperature (SST) gradient has strengthened (*3*). The oceanic and atmospheric drivers of this cooling are under investigation (*4*), but not fully understood. Further complicating the issue, climate models notoriously struggle to simulate the observed eastern Pacific cooling, and it is likely that this model-observation discrepancy originates partly from systematic model biases, potentially in addition to internal variability (*2*).

The Pacific SST pattern exerts a dominant control on the atmospheric general circulation (*5*), tropical cyclones (*6*), clouds (*7*), global radiative feedbacks, climate sensitivity (*8*–*12*), and patterns of precipitation (*13*). Therefore, understanding the processes that shape the Pacific SST pattern, including the recent cooling, is a central puzzle in climate science. It is crucial for interpreting past and predicting future climate change (*6*, *14*), and imperative for mitigation and adaptation efforts (*15*).

A hint of Pacific cooling can be seen in many models’ fast, transient response to abrupt carbon dioxide (CO_2_) forcing, which includes slight cooling or suppressed warming in the equatorial and eastern Pacific (*16*). To explain this, the ocean dynamical thermostat mechanism is commonly invoked (*4*, *16*–*21*): Climatological upwelling through a strengthened ocean stratification cools the eastern Pacific surface. Currently, this is the only known process that can cause a fast absolute cooling of the eastern Pacific in response to CO_2_ forcing.

Here, we propose an alternative driver of transient eastern Pacific cooling that is not rooted in ocean dynamics. We hypothesize that the land-sea heating contrast, which is most pronounced on fast timescales (*22*), plays the leading role in driving the fast equatorial and eastern Pacific cooling response to CO_2_ forcing. This is further motivated by the fact that the Walker circulation strengthens more in models with stronger land-sea contrast (*23*). The land-sea contrast has been speculated to play an important role for setting the fast temperature and circulation response (*24*, *25*). Yet, its importance has not been fully explored, and it is not listed among the processes controlling the Pacific SST pattern in a recent review (*4*).

To examine the effects of land-sea warming contrasts on the Pacific SST pattern, we study the climate response to localized CO_2_ forcing over land versus over ocean. Localized CO_2_ forcing over land in summer has been found to be responsible for the majority of general circulation and precipitation changes in response to global CO_2_ forcing under fixed SST conditions (*26*, *27*). However, it is currently unknown how the anomalous circulations arising from land forcing affect the SST pattern, which is a gap that this paper addresses.

To separate the effect of land CO_2_ forcing from the response to global CO_2_ forcing, we conduct ensembles of 10-year simulations using the Max Planck Institute Earth System Model 1.2 (MPI-ESM) (*28*), in which the atmospheric CO_2_ concentration is abruptly quadrupled globally (4×CO_2_-ALL), over land only (4×CO_2_-LAND), and over ocean only (4×CO_2_-OCEAN). To substantiate the hypotheses we introduce later, we add simulations with quadrupled CO_2_ concentrations only over Northern Hemisphere (NH) land north of 8°N (4×CO_2_-LAND-NH), only over tropical land between 8°N and 8°S (4×CO_2_-LAND-TR), and only over South America south of 8°S (4×CO_2_-LAND-SA). To assess the relevance of our findings under a more realistic forcing scenario, we also perform 40-year simulations with a 1%-per-year CO_2_ increase over land (1% CO_2_-LAND). The CO_2_ concentration is prescribed only as a function of latitude and longitude, and we do not allow CO_2_ to be transported in the atmosphere or taken up by the ocean. In all figures, we show the ensemble-mean response over a 24-member ensemble (48 members for 1% CO_2_-LAND), which effectively singles out the forced response by suppressing natural variability.

## RESULTS

**Fig. 1.**
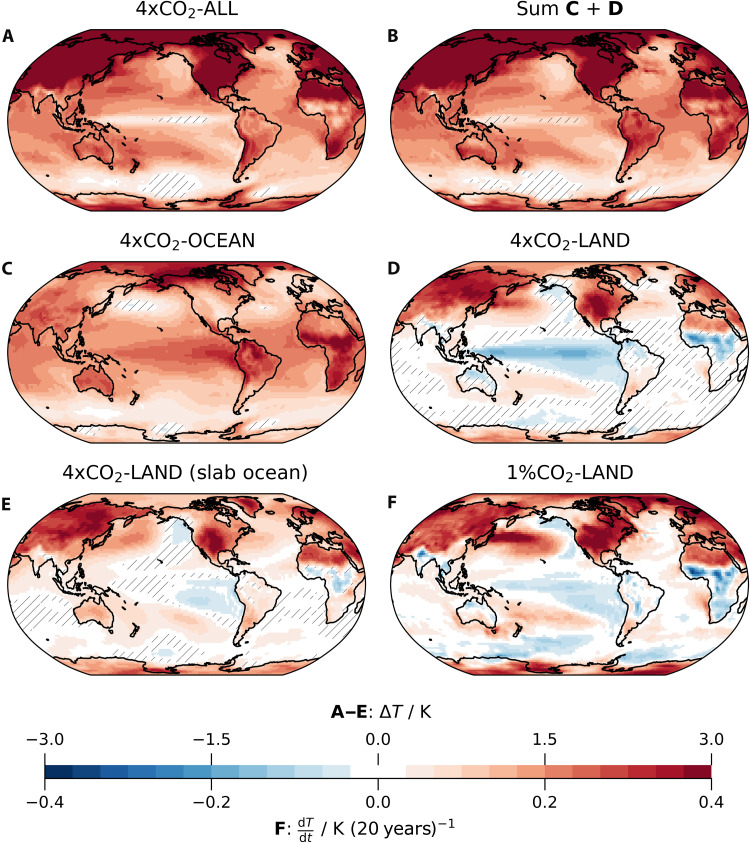
Ensemble-mean response of 2-m air temperatures. (**A**) to (**D**) show the second year of the fully coupled simulations compared to piControl. (**E**) Same as (D), but with a slab ocean instead of a dynamic ocean. Hatched grid points are not significantly different from zero at the 90% confidence level, determined with a two-sided *t* test. (**F**) Twenty-year trend in a simulation with 1%/year CO_2_ increase over land.

**Fig. 2.**
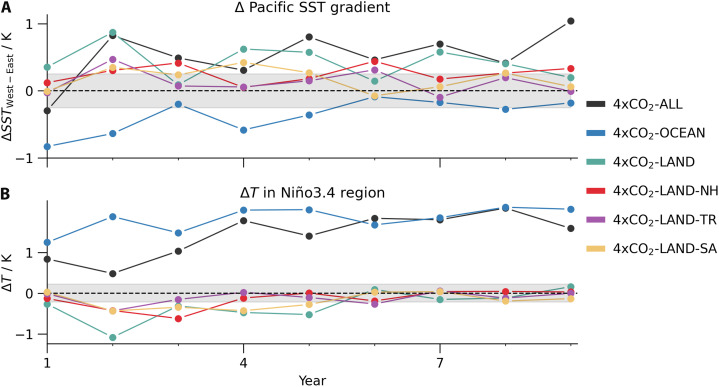
Key metrics of Pacific temperature change patterns. (**A**) Time series of change in Pacific West-East SST gradient (80°E to 150°E versus 155°W to 80°W, both within 5° of the Equator). (**B**) Two-meter air temperature change in the Niño3.4 region. The gray-shaded areas span the SD of a 24-member ensemble obtained from the control simulation.

The initial response to global CO_2_ in 4×CO_2_-ALL is characterized by muted warming in the equatorial Pacific (Fig. 1A), accompanied by an enhanced zonal SST contrast (Fig. 2A). This pattern is well reproduced by the sum of the responses to 4×CO_2_-LAND and 4×CO_2_-OCEAN (Fig. 1, A and B), indicating that the full signal can be meaningfully separated into contributions originating from forcing over land versus ocean.

The 4×CO_2_-LAND and 4×CO_2_-OCEAN simulations reveal that the muted initial response results from compensating effects between El Niño–like warming driven by ocean CO_2_ and La Niña–like cooling driven by land CO_2_ (Fig. 1, C and D). We present the second-year temperature response, when the equatorial and eastern Pacific cooling in 4×CO_2_-LAND, which is the central focus of this study, is most pronounced (up to 1.4 K, blue region in Fig. 1D). We quantify the cooling amplitude based on the temperature changes in the Niño3.4 region, which closely correlate with temperatures in the southeastern Pacific. The absolute cooling in the Niño3.4 region peaks at about 1 K in the second year, remains significant through year 5, but is eventually outpaced by the mean warming within the first decade (green line in Fig. 2B). Nonetheless, the relative coolness of the eastern Pacific compared to the tropical mean persists through the first decade (figs. S1 and S2). Consistently, the West-East temperature gradient in the tropical Pacific, a key metric controlling the Walker circulation response (*5*), remains strengthened throughout the first decade (green line in Fig. 2A). This relative cooling of the eastern compared to the western Pacific SST is a known transient response to abrupt 4×CO_2_ forcing in many models that contributed to the Coupled Model Intercomparison Project (CMIP) (*16*, *24*). However, our regional CO_2_ experiments reveal that land CO_2_ is the primary driver of the increase in zonal SST contrast and that it even leads to an absolute cooling of the eastern Pacific (Fig. 2B).

The absolute cooling in response to land CO_2_ is evident even when using a motionless slab ocean instead of a full dynamic ocean (Fig. 1E), although the amplitude is weaker and the response is confined to the eastern Pacific. This result points toward the ocean’s role of amplifying (but not initiating) the cooling seen in 4×CO_2_-LAND, which we will discuss later.

Some might question the importance of the cooling induced by land CO_2_, as it only emerges during the first 5 years in 4×CO_2_-LAND. However, in the transiently forced 1% CO_2_-LAND simulation, the cooling trend persists for about 20 years (Fig. 1F), indicating that the effects seen in 4×CO_2_-LAND are relevant on decadal timescales under more realistic scenarios. Hence, amplifying the land-sea contrast produces a pronounced cooling in the eastern and equatorial Pacific. We infer that land-sea contrasts are a key driver of the fast cooling response. The absolute cooling can cease for two reasons: first, if the land-sea contrast becomes smaller (e.g., after CO_2_ concentrations plateau), and second, when the forcing persists, but the ocean mean warming outpaces the cooling, as the ocean accumulates more and more heat content over time. In our 1% CO_2_-LAND simulation, this is the case after roughly 20 years.

**Fig. 3.**
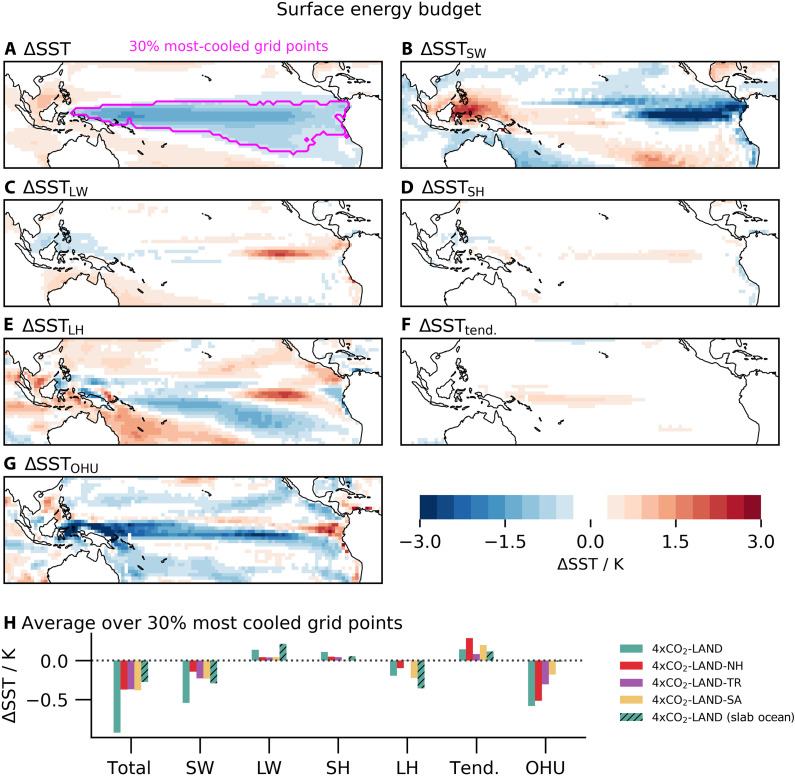
Ocean surface heat budget decomposition. (**A**) The total tropical SST change in the second year of 4×CO_2_-LAND. (**B** to **G**) Contributions to SST changes from changes in (B) shortwave; (C) longwave; (D) sensible heat; (E) latent heat due to changes in wind speed, near-surface relative humidity, or air-surface temperature differences; (F) the tendency term; and (G) ocean heat uptake and oceanic advection (OHU). (**H**) shows the contribution from each component in each simulation, averaged over the 30% most-cooled tropical Pacific grid points in 4×CO_2_-LAND [magenta outline in (A)].

The main goal of this paper is to explain how adding heat over land leads to the transient cooling of the eastern and equatorial Pacific seen in Fig. 1D. A surface heat budget analysis of 4×CO_2_-LAND [Fig. 3; for implementation details, see Materials and Methods or (*29*)] reveals that the cooling is predominantly driven by shortwave (i.e., cloud) effects in the eastern equatorial Pacific, wind-driven cooling in the region of the southerly trade winds, and ocean heat uptake along the equatorial Pacific (Fig. 3, B, E, and G). We infer from this that cloud feedbacks and upwelling on the Equator, as well as enhanced evaporation in the off-equatorial tropics, reminiscent of the wind-evaporation-SST (WES) feedback (*30*), are likely candidates for explaining the origin of the cooling.

With this in mind, we propose three mechanisms that act in concert to elicit the eastern and equatorial Pacific cooling response: (i) a northward intertropical convergence zone (ITCZ) shift due to the preferential NH warming relative to the Southern Hemisphere (SH), (ii) a strengthening of the Walker circulation as a fast response to heating over tropical land, and (iii) a strengthening of the South Pacific subtropical high from Rossby waves due to heating over South America. Our strategy to separate these processes is to use targeted simulations in which the CO_2_ concentration is quadrupled only over parts of the land, such that only one mechanism is directly triggered.

### Northward ITCZ shift

**Fig. 4.**
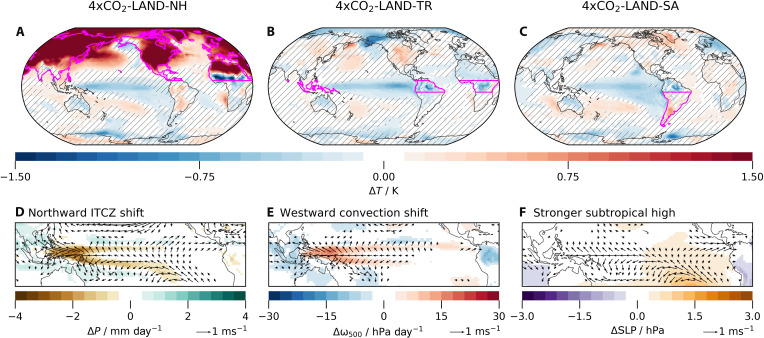
Key quantities to illustrate the three mechanisms that lead to the Pacific cooling. (**A** to **C**) Two-meter air temperature change. Hatched grid points are not significantly different from zero at the 90% confidence level, determined with a two-sided *t* test. The thick magenta outlines indicate the land regions that were forced by quadrupled CO_2_ concentrations (boundaries at 8°N/S). The bottom row shows changes in (**D**) precipitation, (**E**) vertical velocity at 500 hPa, and (**F**) sea level pressure, each representing the dominant mechanism in the respective simulation. The arrows show changes in 10-m wind speed, where changes below 0.2 m s^−1^ are not shown. All panels show the response in the second year, compared to control.

Because there is more land in the NH than in the SH, increasing the CO_2_ concentration only over land introduces more heat into the NH than the SH. Paleoclimate, theory, and modeling studies have consistently shown that hemispherically asymmetric forcing shifts the ITCZ toward the heated hemisphere (*31*–*34*). Thus, land CO_2_ forcing is expected to drive a northward shift of the ITCZ. To isolate the effect of the northward ITCZ shift in 4×CO_2_-LAND, we construct a targeted simulation where the CO_2_ concentration is quadrupled only over land north of 8°N (4×CO_2_-LAND-NH), leaving no local forcing at or south of the Equator. In 4×CO_2_-LAND-NH, the resulting northward ITCZ shift alone induces a cooling patch in the equatorial and southeastern tropical Pacific (Fig. 4A), similar to that seen in 4×CO_2_-LAND, though with only about half the magnitude (Fig. 2B).

A northward shift of the ITCZ is accompanied by a strengthened southern cross-equatorial Hadley cell, which is manifested as enhanced southerly trade winds in its lower branch (*33*, *34*). In 4×CO_2_-LAND, southeasterly wind anomalies emerge in the southeastern tropical Pacific (Fig. 4D). These anomalously strong southerly trades trigger a WES feedback (*30*, *35*, *36*), in which enhanced evaporation cools the eastern Pacific surface, further strengthening the trade winds and reinforcing the cooling through a positive feedback loop. In addition, the strengthened equatorial easterlies drive enhanced upwelling, contributing further to equatorial cooling. The resulting surface cooling promotes an increase in low clouds over the eastern equatorial Pacific, amplifying the cooling via shortwave cloud feedback. Overall, the dominant processes contributing to equatorial cooling involve changes in ocean heat uptake, latent heat flux, and shortwave radiation (red bars in Fig. 3H).

### Westward convection shift

In response to global CO_2_ forcing, the zonal SST gradient initially increases, accompanied by a strengthening of the Walker circulation (Fig. 2A and fig. S8). This fast response to CO_2_ forcing has been identified in many (but not all) CMIP models (*16*, *24*) and has been related to land-sea warming contrasts (*23*, *37*). Accordingly, our 4×CO_2_-LAND simulation exhibits a much stronger initial strengthening of the Walker circulation compared to 4×CO_2_-ALL, as the land-sea warming contrast is amplified (fig. S8). To further isolate the effect of the strengthened Walker circulation, we design an experiment in which only the zonally asymmetric heating is imposed locally to directly perturb the Walker circulation. In this simulation (referred to as 4×CO_2_-LAND-TR), CO_2_ concentrations are quadrupled only over the tropical land between 8°S and 8°N. We find the same cooling patch as in 4×CO_2_-LAND (Fig. 4B), albeit weaker by about 60% (Fig. 2B).

In the tropical atmosphere, the main balance is between diabatic heating and vertical motions. Therefore, radiative heating from CO_2_ over tropical land induces upward motion over the Maritime Continent, shifting the convection away from the western Pacific toward the Maritime Continent (Fig. 4E). A westward circulation shift triggers Kelvin waves (*38*, *39*), which lead to anomalous easterlies along the Equator (arrows in Fig. 4E), initiating the Bjerknes feedback reminiscent of La Niña. The intensification of equatorial easterlies enhances upwelling (purple bars in Fig. 3H), making equatorial coupled atmosphere-ocean dynamics the primary driver of tropical Pacific cooling in 4×CO_2_-LAND-TR. The surface cooling further promotes low cloud formation, with associated shortwave cloud radiative feedback amplifying the cooling.

### Strengthening subtropical highs

The last mechanism builds on the fact that the land-sea contrast strengthens the Pacific subtropical anticyclones. Atmospheric heating causes high pressure and descent to its west, and the subtropical anticyclones are partly the dynamic response to heat release in the free troposphere over subtropical land (*40*–*43*). In 4×CO_2_-LAND, there is anomalous diabatic heating over land due to the increased CO_2_ concentration (Fig. 1A), and it has been recognized that this type of heating strengthens and shifts the subtropical highs over the ocean westward (*26*, *43*–*46*). This would strengthen the equatorward winds on the eastern flanks of the subtropical anticyclones, leading to enhanced evaporation and advection of cold air from subpolar to subtropical latitudes, amplified by the WES feedback, eventually cooling the eastern Pacific. This mechanism, but with opposite sign, has been used to explain why a strengthened Aleutian low leads to a warming of the tropical Pacific (*47*).

To test the implications of this mechanism for the Pacific SST pattern, we create a simulation in which the CO_2_ concentration is quadrupled only over South American land south of 8°S (4×CO_2_-LAND-SA). This targeted experiment successfully isolates the subtropical high mechanism without activating the northward ITCZ shift mechanism, because the NH is not heated. We find a strengthened South Pacific subtropical high pressure system with anomalous southeasterly winds on its eastern flank (Fig. 4F). Consistent with the expectations, 4×CO_2_-LAND-SA produces a cooling patch in the equatorial and eastern Pacific Ocean similar to that in 4×CO_2_-LAND (Fig. 4C), albeit with an amplitude reduced by about 60% (Fig. 2B). This cooling is primarily driven by enhanced latent heat flux due to intensified southerlies associated with the strengthened subtropical high. Surface cooling promotes an increase in low cloud cover, further amplifying the cooling through shortwave cloud feedback (yellow bars in Fig. 3H). Enhanced upwelling associated with intensified equatorial easterlies also contributes to the cooling response. In contrast, forcing only Australia and the Maritime Continent south of 8°S with quadrupled CO_2_ concentrations does not produce a Pacific cooling; hence, we emphasize the importance of the heating to the east of the subtropical anticyclone in driving its intensification.

It is worth noting that the surface of South America remains relatively cool in 4×CO_2_-LAND-SA (Fig. 4C) despite the imposed local CO_2_ forcing. Temperature and atmospheric heating are not always directly linked. In 4×CO_2_-LAND-SA, it is the additional atmospheric diabatic heating over land from the increased CO_2_ concentration that triggers a Rossby wave strengthening the subtropical highs [Fig. 4F; (*26*)]. Similarly, for the ITCZ shift mechanism, the ITCZ does not necessarily shift toward the warmer hemisphere, but rather toward the hemisphere with stronger atmospheric heating (*48*).

### Interactions between different mechanisms

Although each regional forcing experiment is designed to isolate a specific mechanism, tightly coupled ocean-atmosphere feedbacks are rapidly activated, leading to a convergence of the dominant response components across the different experiments (Fig. 3H). For example, the northward ITCZ shift and strengthened Walker circulation, which we identify as drivers of the southeastern Pacific cooling, also arise in response to southeastern Pacific cooling (*35*, *49*, *50*). The zonal and meridional SST gradients are related, linking the westward shift of convection and the northward ITCZ shift mechanisms (*6*, *51*). Consistent with this, they appear in all land-forced simulations (figs. S3 and S4). Similarly, the subtropical highs strengthen not only in response to 4×CO_2_-LAND-SA, but also as a consequence of ITCZ shifts (*52*).

The surface heat budget decomposition averaged over the 30% most-cooled grid points for each targeted simulation (Fig. 3H) reveals that the dominant contributions are broadly similar across these simulations. In all experiments, intensified southerly winds initiate the WES feedback, enhancing surface cooling; strengthened equatorial easterlies drive enhanced upwelling; and the resulting surface cooling promotes low cloud formation, amplifying the cooling response through shortwave cloud feedback. Given this tight coupling, our regional forcing experiments are instrumental in identifying the actual drivers that initiate these feedback loops.

To further examine the interactions between different processes, we turn to simulations in which the atmosphere-ocean coupling is partly disabled by coupling the atmosphere to a motionless slab ocean instead of a dynamic ocean. In the slab-ocean simulations, the feedbacks that amplify the cooling in the central and western Pacific such as the Bjerknes feedback and equatorial upwelling are suppressed, while the WES feedback, which affects mostly the trade wind alley, remains active. Hence, we still find a slight cooling in the southeastern Pacific in the slab-ocean simulations (Fig. 1E and figs. S6 and S7), albeit weaker than in the dynamic ocean simulations and confined to the eastern basin without extending into the central or western Pacific. The terms of the surface energy budget that lead to the cooling differ in magnitude from the simulation with the full ocean model, in particular because the dynamic ocean response is missing, but their signs are the same (compare green hatched and nonhatched bars in Fig. 3H). This indicates that the initial cooling is atmosphere driven. However, coupled air-sea processes, such as wind-driven upwelling and the Bjerknes feedback, are crucial to amplify the cooling and to communicate it across the Pacific basin.

## DISCUSSION

This paper’s key message is that land-ocean heating contrasts shape the fast response to 4×CO_2_ forcing, particularly the cooling of the equatorial and eastern Pacific Ocean. Because the cooling can persist for around 20 years in 1% CO_2_-LAND, we infer that these mechanisms operate also on timescales that are relevant for the recent-past model-observation discrepancy. The colder eastern Pacific in observations compared to models (*2*) is qualitatively similar to the response to 4×CO_2_-LAND [compare our Fig. 1D to figure 1E in (*2*) with the caveat that potentially some of the model-observation difference shown in their figure 1E is due to internal variability]. If the mechanisms we describe, which translate the forcing over land to a cooling of the eastern Pacific, are misrepresented, then that may contribute to explaining why models lack cooling in the equatorial and eastern Pacific Ocean.

**Fig. 5.**
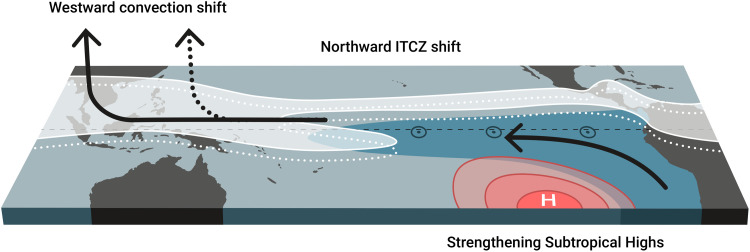
Northward ITCZ shift, westward convection shift, and strengthened subtropical highs cause the Pacific cooling response to land warming. The arrows represent the shifted convection and the strengthened trade winds, while the circles represent increased upwelling. The figure was created by Y. Schrader, used with permission.

Motivated by the model-observation discrepancy in eastern Pacific SST, we identify three mechanisms that drive Pacific cooling, which are illustrated in Fig. 5: a northward ITCZ shift due to heating over NH land, a westward shift of convection with strengthening equatorial easterlies due to the heating over tropical land, and a strengthening subtropical anticyclone due to heating over South America. One obvious interpretation for the origin of the model-observation discrepancy is that models might underestimate the land-sea contrast. This would trigger the mechanisms we describe insufficiently. Our results highlight the need to scrutinize uncertainties in land models, while previous studies have largely focused on ocean-origin mechanisms to explain the cooling.

However, the representation of land-sea contrasts is not the only possible origin of the model-observation discrepancy. Models might misrepresent the balance between the ocean-only and land-only processes. For a climate model to correctly simulate the eastern Pacific cooling, all the subprocesses involved in the mechanisms we identify must be accurately represented. This includes the positive low cloud feedback over the subtropical eastern Pacific subsidence regions, which is a known source of uncertainty in climate models (*53*–*55*) and has been shown to be important in the positive feedback loop of amplifying subtropical Pacific temperature changes and communicating them to the tropical Pacific (*35*, *36*, *54*, *56*). Cloud feedbacks are also important for the SST pattern on the Equator (Fig. 3B). Ocean dynamical processes such as upwelling need to be properly simulated (*35*) because they determine ocean heat uptake, which we show to be essential (Fig. 3G). Furthermore, the notorious double ITCZ model bias (*57*) has been shown to hamper the correct simulation of the SST pattern (*49*, *58*) and directly affects the ITCZ shift mechanism.

MPI-ESM is among the models that most closely reproduce the historical warming pattern, including the enhanced zonal SST gradient (*2*). This could be because this model properly captures the land-driven cooling response, while some other models might not exhibit the same extent of cooling response to CO_2_ forcing over land. It would be interesting to study the response to CO_2_ forcing only over land in other models in the future.

Our results challenge the prevailing notion that the ocean dynamical thermostat mechanism drives the transient Pacific cooling (*16*, *17*, *20*, *21*); instead, they recast it as a feedback. The thermostat mechanism is centered around vertical motion in the ocean without relying on complex atmospheric dynamics and predicts a cooling of the eastern equatorial Pacific in response to uniform forcing to the ocean. Hence, if it were the driving mechanism, we would expect to see cooling in 4×CO_2_-OCEAN. Instead, we find enhanced eastern and equatorial Pacific warming (Fig. 1C). Moreover, we find cooling in the eastern equatorial Pacific and a strengthened SST gradient in the land-forced simulations, even when coupled to a slab ocean where the ocean dynamic thermostat mechanism is inactive (Fig. 1E and fig. S7). The strengthened SST gradient persists in the land-forced simulations with dynamic and slab ocean throughout the 10 simulated years without signs of weakening. This indicates that atmosphere-driven processes suffice to sustain a multidecadal strengthening of the Pacific Equatorial SST gradient, in contrast to the prevailing view that this can only be achieved by the ocean dynamical thermostat mechanism. We do not argue that the dynamic ocean plays no role for setting the SST pattern. We find an elevated role for the ocean dynamics term in the surface energy budget (Fig. 3G). A heat budget analysis of the upper ocean reveals a dominant cooling contribution from the vertical and meridional advection terms, indicating a critical role for dynamical ocean cooling (fig. S9 and the Supplementary Text). However, the presence of cooling in the slab-ocean simulation and the absence of cooling in the ocean-forced simulation suggest that the ocean dynamic thermostat mechanism only amplifies the atmosphere-induced cooling. All mechanisms we find are initiated by the atmosphere, and the ocean acts as a feedback.

One might argue that our model may simply not capture the ocean thermostat response. For example, a too diffuse thermocline due to limited vertical resolution might prevent a proper representation of the ocean dynamical thermostat mechanism (*59*). However, MPI-ESM has been classified as a model (perhaps even the quintessential model) representative of an “ocean-thermostat”–like response to CO_2_ forcing (*20*). Therefore, we interpret the absence of the expected thermostat-like response in 4×CO_2_-OCEAN to be no model deficiency, but rather a sign of the thermostat mechanism arising only as a feedback to external perturbations (*60*).

The eastern and equatorial Pacific cooling we find is transient in nature. It may last for decades (Fig. 1F), but as the land-sea contrasts diminish and ocean heating increases with time, the cooling of the eastern Pacific will eventually be outpaced by the ocean heating. Hence, our analysis, combined with the assessment of Watanabe *et al.* (*4*), suggests that the currently observed pattern of eastern Pacific cooling will eventually flip toward a strengthened eastern Pacific warming. This prediction will not hold if other unknown mechanisms take over and allow the cooling to persist longer. It might fail if the positive feedbacks enhancing the eastern Pacific cooling are severely underestimated in models, or due to a sustained remote cooling influence on the tropical Pacific from the Southern Ocean (*4*, *54*), which is not accounted for in our study.

## MATERIALS AND METHODS

### Model

We use the MPI-ESM 1.2 (*28*) with the atmosphere model ECHAM6 (*61*). Our main analysis refers to simulations with the atmosphere component coupled to the dynamic ocean model MPIOM (*62*), but we later compare to simulations with a mixed-layer ocean with a depth of 50 m. The atmosphere has a horizontal resolution of 1.875° on 47 vertical levels, and the dynamic ocean is simulated on a bipolar grid with 1.5° nominal resolution and 40 vertical levels.

### Forcing

In our simulations, we prescribe CO_2_ concentrations as a function of longitude and latitude. We do not allow CO_2_ to be transported, taken up, or released by the ocean, or to interact with vegetation. Hence, our simulations only represent the purely radiative effects of CO_2_ without the plant-physiological effects, and CO_2_ is constant in time (except in 1% CO_2_-LAND), but not in space. We apply the following forcings: 4×CO_2_-ALL quadrupled CO_2_ everywhere; 4×CO_2_-LAND quadrupled CO_2_ over all land; 4×CO_2_-OCEAN quadrupled CO_2_ over all ocean; 4×CO_2_-LAND-NH quadrupled CO_2_ over NH land north of 8°N; 4×CO_2_-LAND-TR quadrupled CO_2_ over tropical land within 8°S to 8°N; and 4×CO_2_-LAND-SA quadrupled CO_2_ concentrations over South American land south of 8°S. In addition, we perform a simulation with CO_2_ forcing only over land (as in 4×CO_2_-LAND), but instead of abruptly increasing the CO_2_ concentrations, we increase them by 1% per year (1% CO_2_-LAND).

### Fully coupled simulations

Starting from a 500-year spinup run, we perform one 600-year control simulation with a dynamic ocean and a preindustrial CO_2_ concentration of 280 parts per million (piControl). We branch off simulations every 25 years from this control run, leading to 24 ensemble members for each type of forcing. To increase the signal-to-noise ratio, we perform another 24 ensemble members for 1% CO_2_-LAND, where the branching dates are shifted by 12 years with respect to the initial branching dates. All shown results are the differences between the ensemble-mean forced and the 600-year-mean piControl simulation.

### Slab-ocean simulations

Starting from a 50-year spinup run with a slab ocean, we run a slab-ocean control simulation over 24 years. We branch off one simulation in each year, leading to 24 ensemble members for each type of forcing. All results we show for the slab-ocean simulations are the differences between the ensemble-mean forced and the 24-year-mean control simulation.

### Surface heat budget decomposition

For the surface heat budget analysis shown in Fig. 3, we follow (*29*), with the important difference that our simulations are not in equilibrium. Hence, we have to include a tendency term. The mixed layer budget is

$$\rho c_{p}h\frac{\text{∂}T^{\prime }}{\text{∂}t}=Q_{\text{SW}}^{\prime }+Q_{\text{LW}}^{\prime }+Q_{\text{SH}}^{\prime }+Q_{\text{LH}}^{\prime }+\text{OH}U^{\prime }$$ (1)

where $\rho c_{p}h$ is the heat capacity of the mixed layer, T is the mixed layer temperature (approximately taken to be the SST), Q is the surface flux, primes denote anomalies, and subscripts SW, LW, SH, and LH denote contributions from shortwave, longwave, sensible, and latent heat fluxes, respectively. OHU is ocean heat uptake, which also includes horizontal transports within the mixed layer. Parameterizing the latent heat anomaly as the sum of a term that depends on temperature and a term that depends on all other influences on latent heat flux (wind speed, near-surface relative humidity, and air-surface temperature differences) $Q_{\text{LH}}^{\prime }=\alpha Q_{\text{LH}}T^{\prime }+Q_{\text{LH},\text{other}}^{\prime }$, taking α=0.06 K^−1^ from the Clausius-Clapeyron equation, and assuming that all other terms depend much less strongly on temperature than $Q_{\text{LH}}^{\prime }$, one can rearrange Eq. 1 to

$$T^{\prime }=\frac{-Q_{\text{SW}}^{\prime }-Q_{\text{LW}}^{\prime }-Q_{\text{SH}}^{\prime }-Q_{\text{LH},\text{other}}^{\prime }-{\text{OHU}}^{\prime }+\rho c_{p}h\frac{\text{∂}T^{\prime }}{\text{∂}t}}{\alpha Q_{\text{LH}}}$$ (2)

In the Results, we drop the subscript “other” when describing the latent heat contribution due to changes in winds, near-surface relative humidity, and air-surface temperature differences. Each term in Eq. 2 has the unit K and can be identified as the temperature change due to changes in SW, LW, SH, LH, ocean heat uptake (including horizontal temperature advection), and the tendency term, respectively. To calculate the tendency term, we take the density of seawater ρ=1025 kg m^−3^, the specific heat capacity of seawater $c_{p}=3985$ J kg^−1^ K^−1^, and the mixed layer depth h approximately as the depth of the 20°C isotherm. The ocean heat uptake term is diagnosed as the residual.
