# Supplementary material for: Heating the land cools the eastern and equatorial Pacific
Source: Sci Adv. 2026 Jun 3;12(23):eaeb7004. doi: 10.1126/sciadv.aeb7004 (PMC13232552; doi:10.1126/sciadv.aeb7004)
Supplement: Supplementary file 1 — Supplementary Text Figs. S1 to S9 [file sciadv.aeb7004_sm.pdf]

Science Advances
AAAS

Supplementary Materials for

Heating the land cools the eastern and equatorial Pacific

Moritz Günther *et al.*

Corresponding author: Moritz Günther, moritz.guenther@mpimet.mpg.de

Sci. Adv. **12**, eaeb7004 (2026)
DOI: 10.1126/sciadv.aeb7004

This PDF file includes:

Supplementary Text
Figs. S1 to S9

Supplementary Text accompanying Fig. S9

We disentangle the contributions to the temperature change in the Niño3.4 region with an upper-ocean heat budget. The temperature tendency (expressed as a heat flux in $W\,m^{-2}$) is the sum of surface fluxes (neglecting small leakage of SW radiation into the deeper ocean layers), the tendency term, resolved advection, parameterized advection, and parameterized dianeutral mixing (*61*). The advection terms are computed from the monthly-mean temperature and velocity fields (with second-order finite differences for the horizontal temperature gradient and a forward difference for the vertical temperature gradient), the remaining terms are directly available as model output. These terms are integrated up to 27 m depth in the Niño3.4 region, which is above the climatological thermocline. We also compute a residual that is non-negligible due to regridding, neglecting the faster-than-monthly time scales, and a potential leakage of SW radiation below 27 m.

Fig. S9 reveals that the main balance to the positive surface fluxes is the cooling from advection terms. We interpret both the meridional and vertical contribution to stem from upwelling, because the upwelled water is transported poleward. The upwelling-related cooling strengthens in $4\times CO_2$-ALL and $4\times CO_2$-LAND, but weakens in $4\times CO_2$-OCEAN. Despite the residual being non-negligible, it is clear that the advection terms are the dominant contribution to the cooling in $4\times CO_2$-LAND, and also to the heating in $4\times CO_2$-OCEAN. It is furthermore interesting to note that the Niño3.4 region is subject to the same surface flux in $4\times CO_2$-LAND and $4\times CO_2$-OCEAN, but reacts with cooling in the former and heating in the latter.

In summary, this analysis confirms the interpretation from Fig.3g,h that upwelling strongly contributes to enhancing the cooling in the Eastern Pacific.

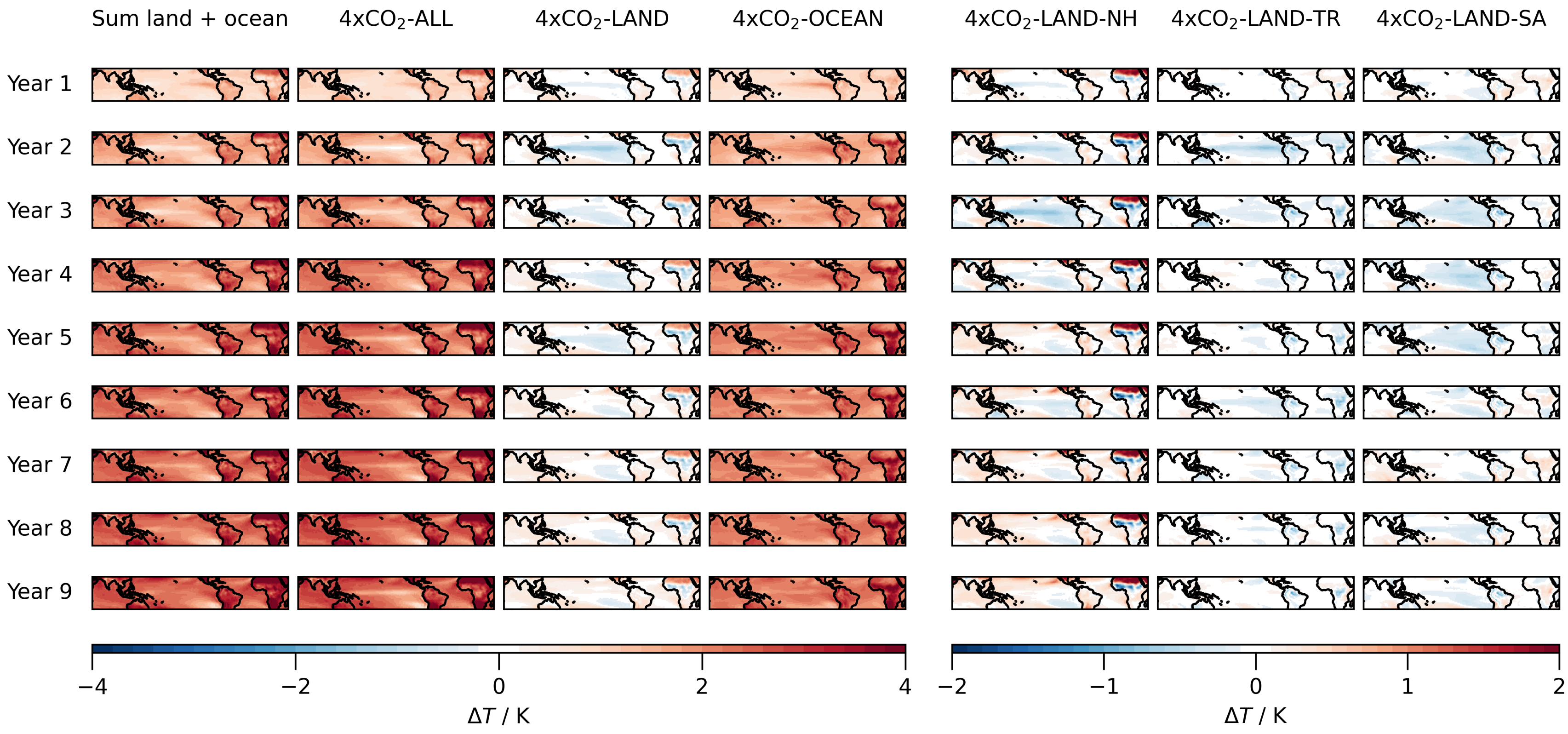

Figure S1: **2-m-air temperature change in the tropics, compared to control.** Since the signal is smaller in the simulations in which not at least the total land surface is forced, we use a differently scaled colorbar for showing 4×CO_2-LAND-NH, 4×CO_2-LAND-TR and 4×CO_2-LAND-SA.

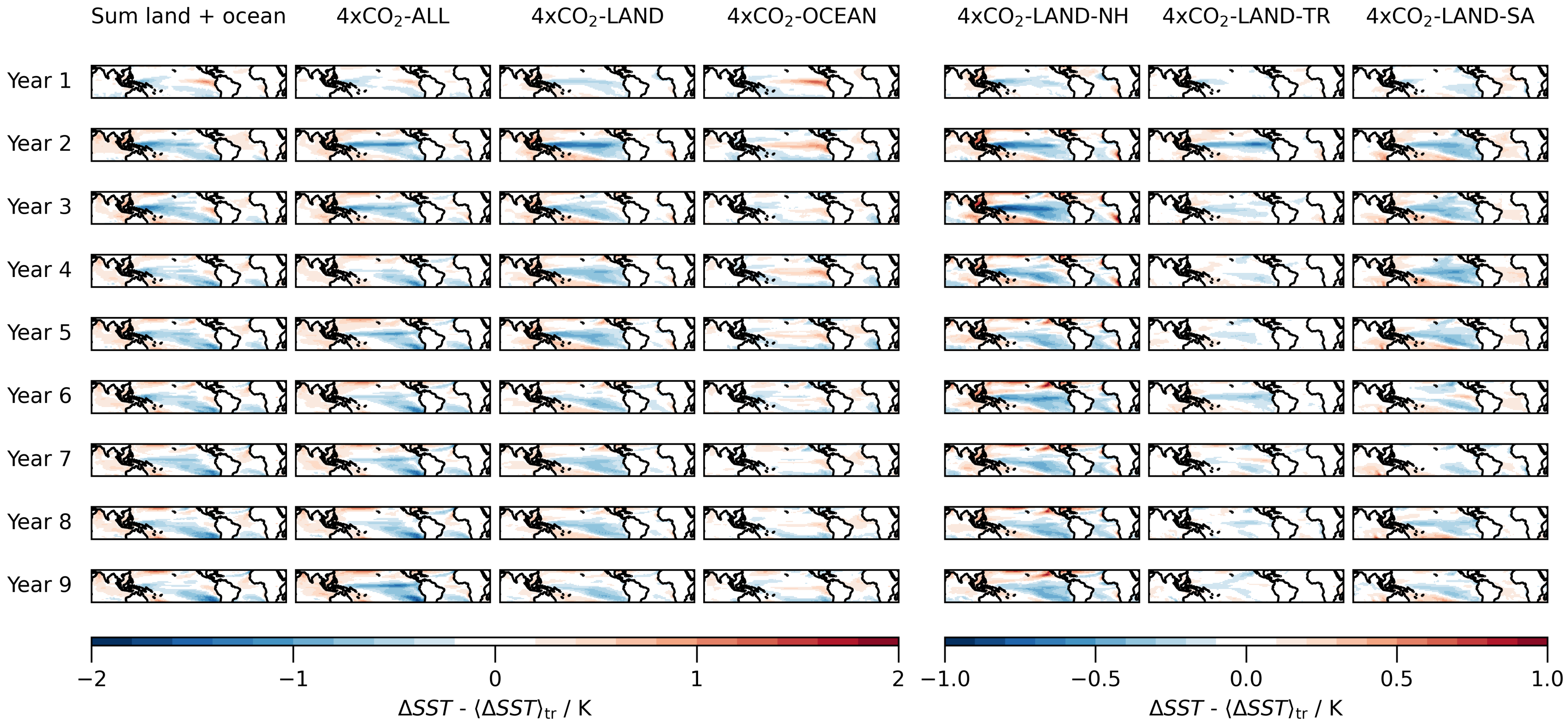

Figure S2: **SST change in the tropics with the tropical mean removed, compared to control.** Since the signal is smaller in the simulations in which not at least the total land surface is forced, we use a differently scaled colorbar for showing 4×CO_2-LAND-NH, 4×CO_2-LAND-TR and 4×CO_2-LAND-SA.

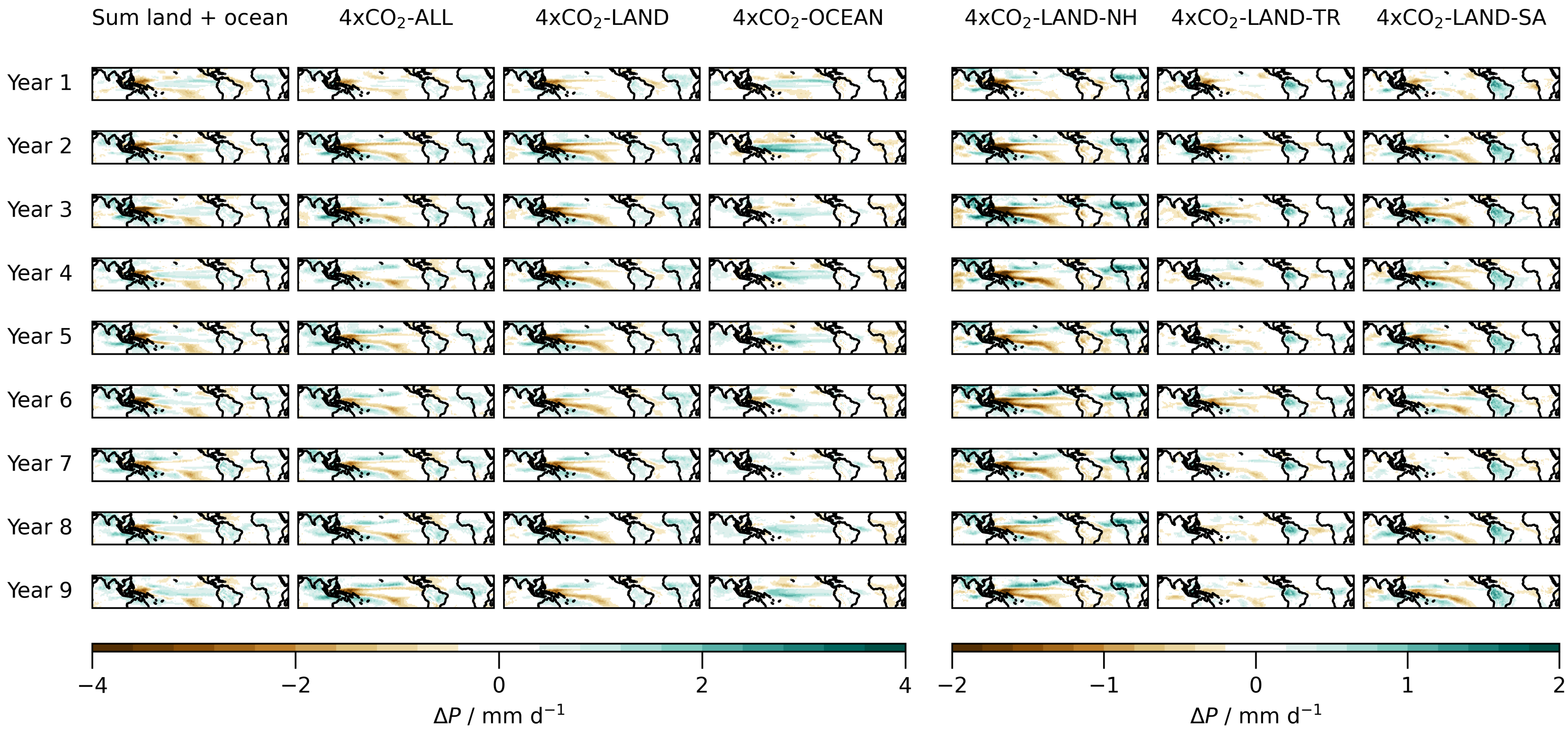

Figure S3: **Precipitation change in the tropics, compared to control.** Since the signal is smaller in the simulations in which not at least the total land surface is forced, we use a differently scaled colorbar for showing 4×CO_2-LAND-NH, 4×CO_2-LAND-TR and 4×CO_2-LAND-SA.

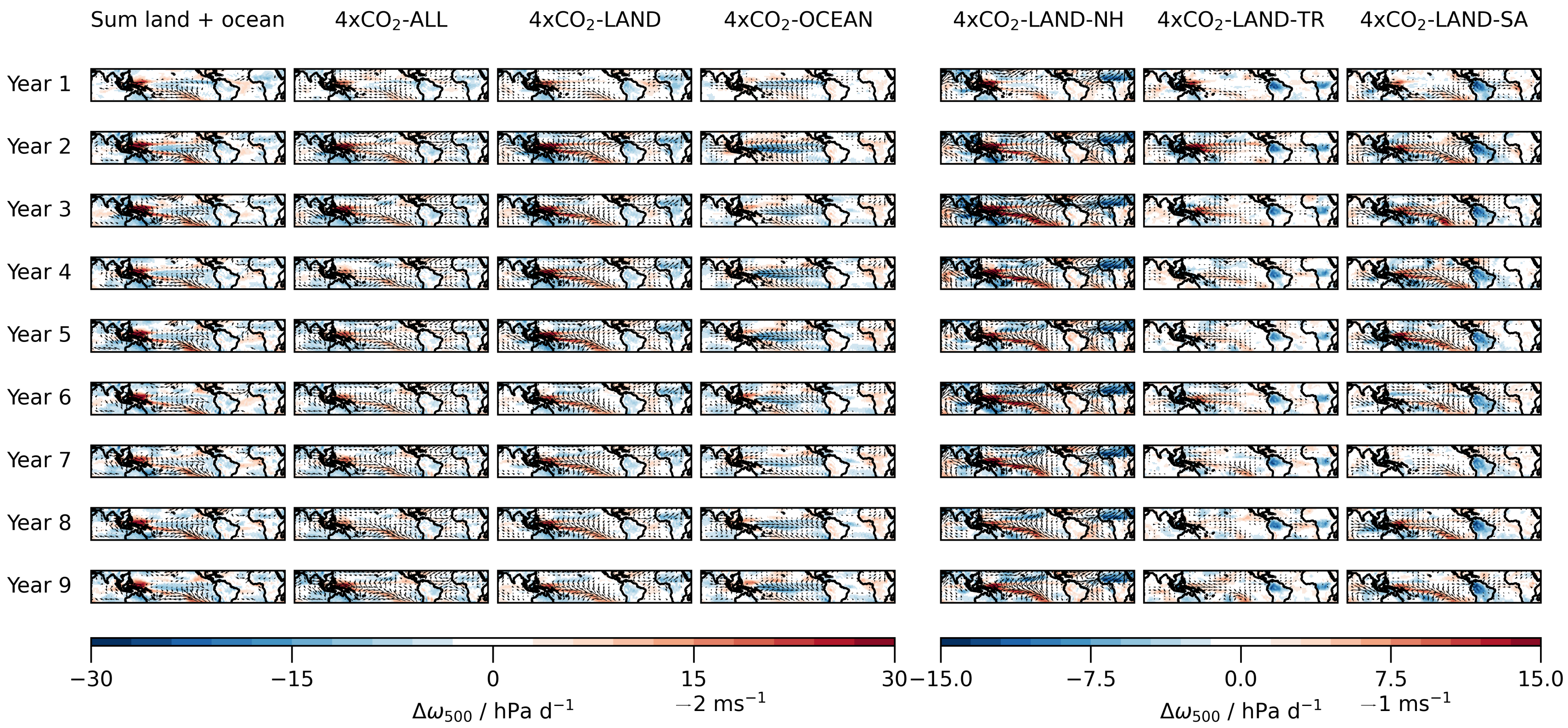

Figure S4: **Changes in vertical velocity (color, positive downward) and horizontal winds (arrows) in the tropics, compared to control.** Since the signal is smaller in the simulations in which not at least the total land surface is forced, we use a differently scaled colorbar and vectors for showing 4×CO_2-LAND-NH, 4×CO_2-LAND-TR and 4×CO_2-LAND-SA.

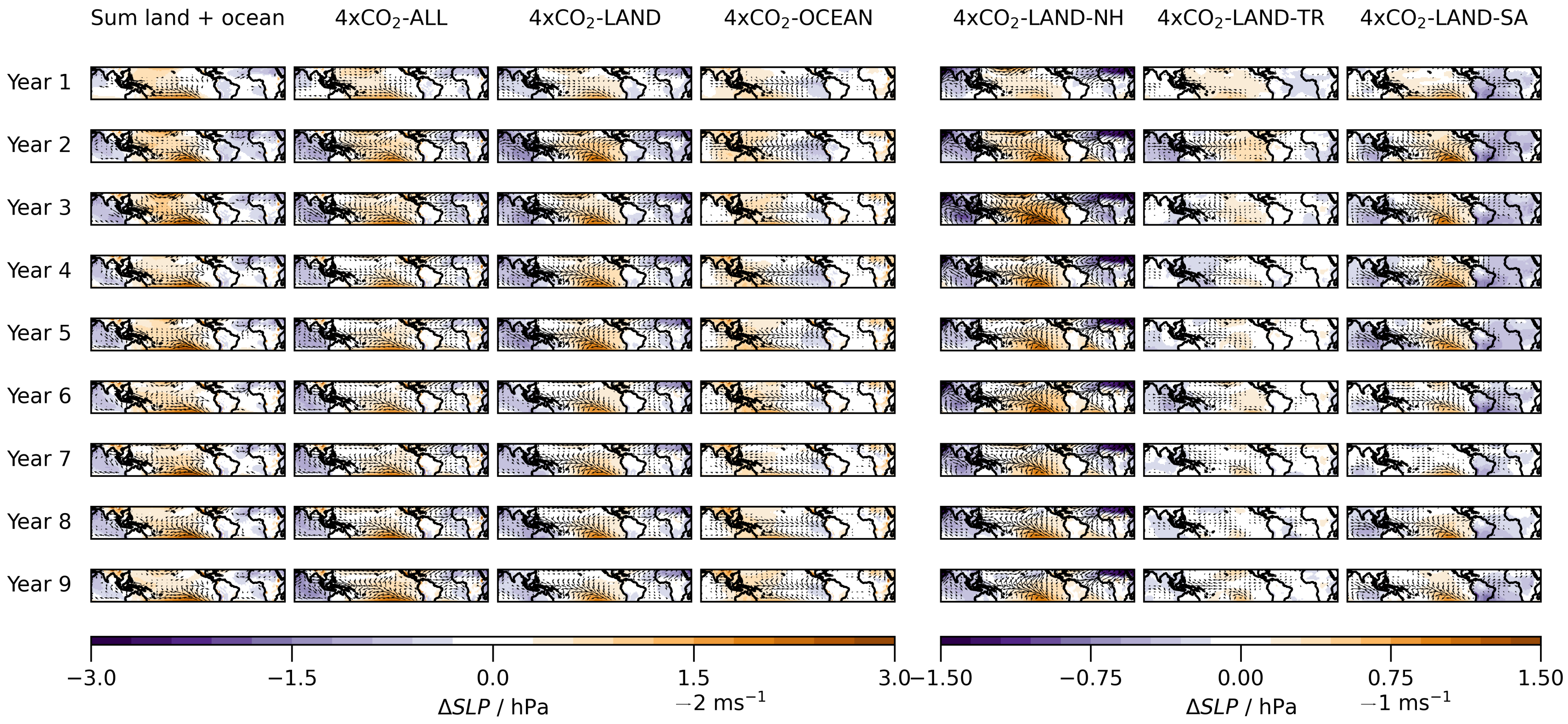

Figure S5: **Changes in sea level pressure (color) and horizontal winds (arrows) in the tropics, compared to control.** Since the signal is smaller in the simulations in which not at least the total land surface is forced, we use a differently scaled colorbar for showing 4×CO_2-LAND-NH, 4×CO_2-LAND-TR and 4×CO_2-LAND-SA.

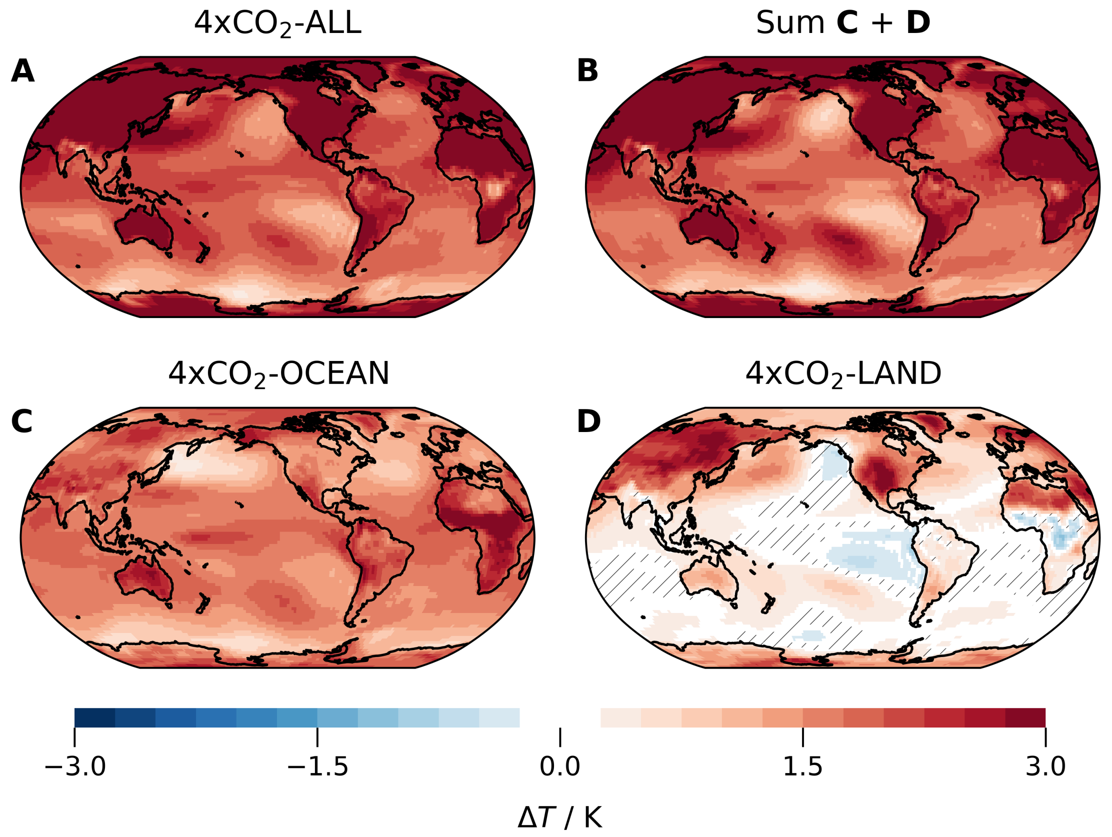

Figure S6: **Ensemble-mean response of 2-m-air temperatures in the second year of the slab-ocean simulations compared to piControl.** Hatched grid points are not significantly different from zero at the 90% confidence level, determined with a two-sided t-test. Same as Fig. 1 from the main manuscript, but for simulations coupled to a slab ocean instead of a full dynamic ocean.

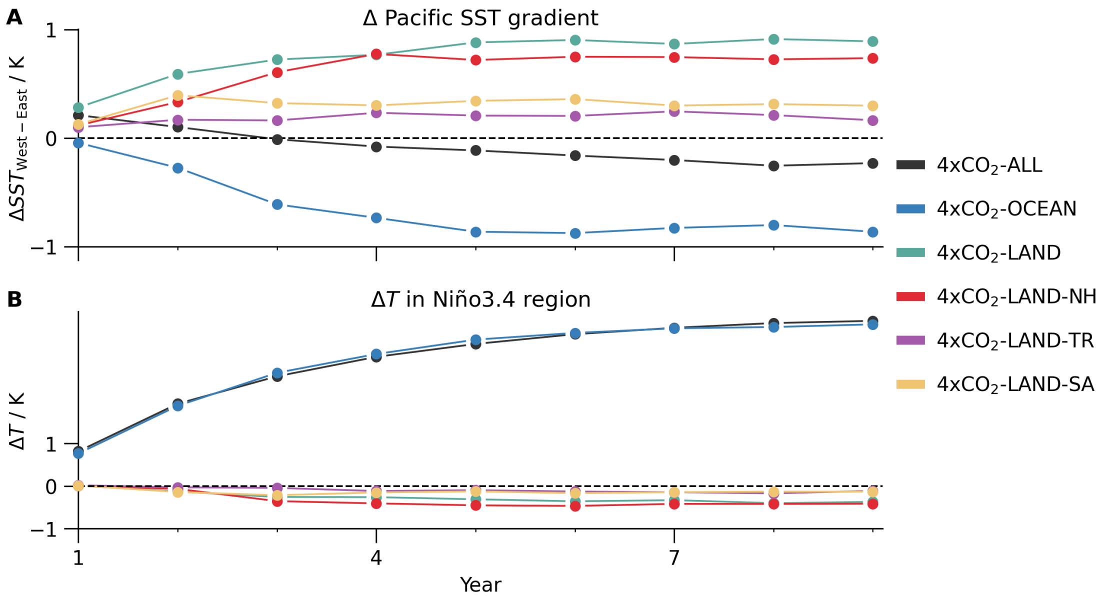

Figure S7: **Key metrics of Pacific temperature change patterns. A** Time series of change in Pacific West-East SST gradient (80°E - 150°E vs 155°W - 80 °W, both within in 5° of the Equator), **B** 2-m-air temperature change in the Niño3.4 region. The gray-shaded areas span the standard deviation of a 24-member ensemble obtained from the control simulation. Same as Fig. 2 from the main manuscript, but for simulations coupled to a slab ocean instead of a full dynamic ocean.

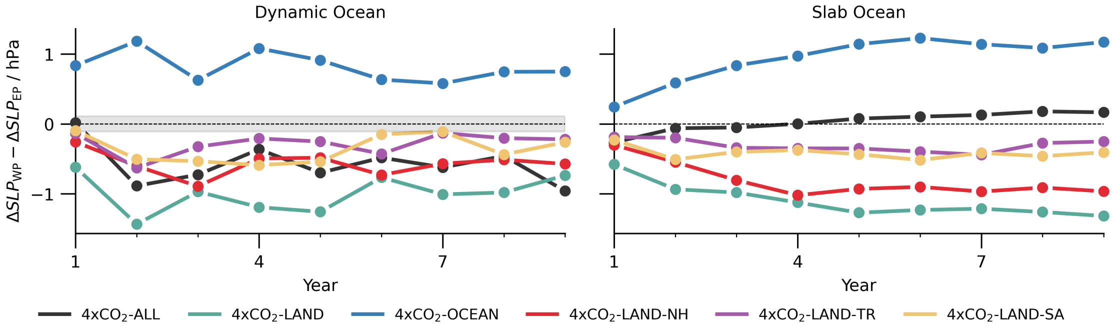

Figure S8: **Changes of the Walker circulation.** The Walker circulation index is computed as defined by (*62*) (Sea level pressure difference between the Western and Eastern Pacific (160°W–80°W vs. 80°E–160°E, both within in 5° of the Equator). The gray-shaded area in **A** spans the standard deviation of a 24-member ensemble obtained from the control simulation

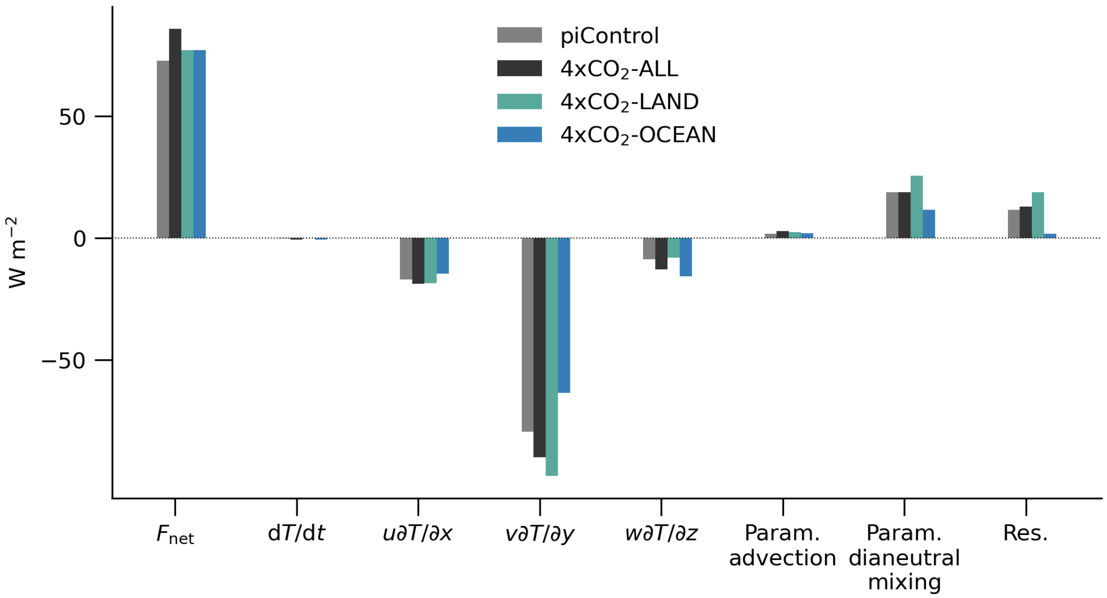

Figure S9: **Upper-ocean heat budget analysis.** All quantities are expressed as heat fluxes and integrated over the upper 27 m of the Niño3.4 region.
